# Serum thioredoxin and lactoferrin in rheumatoid arthritis and their association with rheumatoid factor

**DOI:** 10.5937/jomb0-44758

**Published:** 2025-03-21

**Authors:** Ginka Delcheva, Katya Stefanova, Teodora Stankova, Ana Maneva

**Affiliations:** 1 Medical University of Plovdiv, Faculty of Pharmacy, Department of Medical Biochemistry, Plovdiv, Bulgaria

**Keywords:** lactoferrin, oxidative stress, rheumatoid arthritis, rheumatoid factor, thioredoxin 1, laktoferin, oksidativni stres, reumatoidni artritis, reumatoidni faktor, tioredoksin 1

## Abstract

**Background:**

Thioredoxin (Trx) and lactoferrin (Lf) are multifunctional proteins that are part of the body's antioxidant defence and counteract oxidative tissue damage. Our study aims to investigate Trx and LF levels in the serum of rheumatoid arthritis (RA) patients and establish the association of these proteins with the rheumatoid factor (RF) and other disease markers.

**Methods:**

The study included 114 patients with RA and 42 healthy subjects. Serum concentrations of Trx 1, LF, RF, anti-cyclic citrullinated peptide (anti-CCP) antibodies, Creactive protein (CRP), and IL-6 were determined using commercially available ELISA kits.

**Results:**

Serum thioredoxin 1 levels in RA were significantly higher compared to the control group, 36.4 (29.6-40.2), ng/mL versus 19.0 (16.3-26.8), ng/mL, p<0.0001. Serum lactoferrin levels were elevated in RA compared to the control group. However, the difference was not statistically significant, 579.6 (312.8-947.5) ng/mL versus 519.0 (262.5-928.0) ng/mL. We found substantial negative Trx 1 and LF correlations with rheumatoid factor in RA (r=-0.254, p=0.05 and r=-0.238, p=0.014, respectively). The correlations of thioredoxin and lactoferrin with other disease markers, such as anti-CCP antibodies, DAS28, erythrocyte sedimentation rate (ESR), CRP, and IL6 were not statistically significant. A strong positive correlation between Trx 1 and LF was observed in the study group of RA patients (r=0.519, p<0.0001) but not in the control group.

**Conclusions:**

Thioredoxin and lactoferrin were associated with rheumatoid factor but not with anti-CCP antibodies and systemic disease activity; therefore, the two proteins may serve as new biomarkers for assessing pathological changes and monitoring disease severity and progression in RA.

## Abstract (sr)

**Uvod:**

Tioredoksin (Trx) i laktoferin (Lf) su multifunkcionalni proteini koji su deo antioksidativne odbrane organizma i pomažu u suzbijanju oksidativnog oštećenja tkiva. Naša studija ima za cilj da ispita nivoe Trx i LF u serumu pacijenata sa reumatoidnim artritisom (RA) i uspostavi povezanost ovih proteina sa reumatoidnim faktorom (RF) i drugim markerima bolesti.

**Metode:**

Studija je obuhvatila 114 pacijenata sa reumatoidnim artritisom i 42 zdrave osobe. Koncentracije Trx 1, LF, RF, antitela na ciklični citrulinirani peptid (anti-CCP), Creaktivnog proteina (CRP) i IL-6 u serumu određene su korišćenjem komercijalno dostupnih ELISA kompleta.

**Rezultati:**

Nivoi tioredoksina 1 u serumu kod pacijenata sa RA su bili značajno vi{i u poređenja sa kontrolnom grupom, 36,4 (29,6-40,2) ng/mL naspram 19,0 (16,3-26,8) ng/mL, p<0,0001. Nivoi laktoferina u serumu su bili povećani kod pacijenata sa RA u poređenja sa kontrolnom grupom, ali razlika nije bila statistički značajna, 579,6 (312,8-947,5) ng/mL naspram 519,0 (262,5-928,0) ng/mL. Ustanovili smo značajnu negativnu korelaciju između Trx 1 i LF sa reumatoidnim faktorom kod pacijenata sa RA (r=-0,254, p=0,05 i r=-0,238, p=0,014, respektivno). Korelacije tioredoksina i laktoferina sa drugim marekerima bolesti, kao što su anti-CCP antitela, DAS28, brzina sedimentacije eritrocita (ESR), CRP i IL-6, nisu bile statistički značajne. U grupi pacijenata sa RA primećena je jaka pozitivna korelacija između Trx 1 i LF (r=0,519, p<0,0001), dok takva korelacija nije zabeležena u kontrolnoj grupi.

**Zaključak:**

Tioredoksin i laktoferin su bili povezani sa reumatoidnim faktorom, ali ne i sa anti-CCP antitelima i sistemskom aktivnošću bolesti, stoga ova dva proteina mogu služiti kao novi biomarkeri za procenu patoloških promena i praćenje tezine i progresije bolesti kod reumatoidnog artritisa (RA).

## Introduction

Rheumatoid arthritis (RA) is a progressive autoimmune disease characterized by chronic synovial joint inflammation, progressive erosions, and cartilage destruction [1]. Genetic and environmental factors such as smoking and viral and bacterial infections cause impairment of the immune system and development of RA, whose prevalence is 0.5–2% [2] [3] [4] [5].

The increased production of reactive oxygen species (ROS) and reactive nitrogen species (RNS) is the cause of oxidative stress in RA, followed by oxidative post-translational modifications of proteins such as citrullination [6]. A number of antioxidant enzymes regulate ROS concentration. These are SOD, glutathione peroxidase, glutathione reductase, and catalase. Thioredoxin is a ubiquitous protein with various functions, including DNA synthesis, defence against oxidative stress, cell growth and apoptosis [7] [8]. Trx is also considered an antioxidant enzyme since itsmajor function is maintaining protein thiol groups in the reduced state [6] [7] [8] [9]. Trx expression is induced by inflammation and oxidative stress, which elevates Trx serum levels [10] [11].

Lactoferrin is an iron-binding glycoprotein found on mucosal surfaces, within neutrophils and various cells, and in colostrum, breast milk and digestive juices. Most of Lf in serum is released from the degranulation of neutrophils [12]. Lactoferrin can lower inflammation and protect against oxidative stress, critical mechanisms involved in the progression of various diseases. The protein’s anti-inflammatory activity is explained by its ability to decrease the release of TNF-α and IL-6 [13] [14] [15] [16] [17]. Lactoferrin controls key antioxidant enzymes, and its antioxidant activity contributes to maintaining the balance between ROS generation and the rate of their elimination, thus decreasing oxidative stress and cell and tissue damage. Lactoferrin concentration is strongly influenced by inflammatory processes associated with neutrophil degranulation, leading to Lf secretion [14].

In rheumatoid arthritis, rheumatoid factor is a primary disease-related biomarker. RF is an autoantibody produced by B-cells targeting the Fc region of IgG [18] [19]. RF estimations, together with anti-CCP antibodies, are meaningful in the diagnosis and management of RA patients. It is suggested that anti-CCP antibodies help establish the diagnosis of RA. However, RF estimations are better predictors of disease severity. They are recommended as a prognostic indicator of disease activity and progression [20].

The present work aimed to determine the levels of thioredoxin 1 and lactoferrin in the serum of patients with RA and healthy controls and to establish the association of these proteins with rheumatoid factor and other disease markers.

## Materials and methods

The study population consisted of 114 patients with RA (16 male and 98 female, mean age 58±10 years) admitted to the Rheumatology departments of the University hospitals at the Medical University of Plovdiv. All patients were diagnosed with RA according to EULAR (European League Against Rheumatism) 2010 criteria. The control group consisted of 42 healthy subjects without any history of inflammatory disease (11 male and 31 female, mean age 34±14 years). All patients were receiving NSAIDs and additional therapy as follows: DMARDs (n=83), DMARDs and corticosteroids (n=10), biological agents, DMARDs and corticosteroids (n=4), corticosteroids (n=2), biological agents and DMARDs (n=11), biological agents and corticosteroids (n=3). Patients with RA were distributed in two subgroups according to sex, CRP level and DAS28. CRP value of 8 mg/mL and DAS28 of 5.1 were used as a cut-off point. Patients in the group with DAS28<5.1 had moderate disease activity, and patients with DAS28> 5.1 had high disease activity.

Blood was collected in monovettes without anticoagulants. Thirty minutes after blood collection, the tubes were centrifuged at 3000 g for 10 minutes. The serum was stored at -80°C before analysis. Serum concentrations of thioredoxin 1, ng/mL and lactoferrin, ng/mL, were determined using commercially available ELISA kits (AbFrontier, Seoul, Korea and MyBioSource, San Diego, USA). Serum concentrations of CRP, mg/mL and IL-6, pg/ml were determined with ELISA kits (BioVendor – Laboratorni-medicina, Brno, Czech Republic). RF, U/mL was determined with ELISA kits (Nova Tec Immun-diagnostica, GmbH, Germany) and anti-CCP antibodies, U/mL with ELISA kits (Eurodiagnostica, Sweden). The measurements were performed on ELISA reader HumaReader HS, HUMAN (Wiesbaden, Germany).

Statistical analysis was performed using SPSS software, version 17.0 (SPSS Inc., Chicago, IL, USA). Normal distribution was assessed using the Kolmogorov-Smirnov test. Continuous variables were expressed as mean ± SD or as median and 25th-percentile–75th-percentile. Student’s t-test was used to compare two groups with Gaussian distribution, and the Mann-Whitney U test was used to compare groups with non-Gaussian distribution. Correlations between data were evaluated by calculating Pearson’s or Spearman’s correlation coefficient depending on the distribution of the continuous variables. p<0.05 was considered statistically significant.

## Results

The main demographic and clinical characteristics of the study RA patients are summarized in Table 1.

**Table 1:** Demographic and clinical characteristics of the study RA patients. Data are presented as mean ± SD or median (25th–75th percentile).

Characteristics	
Mean age, years	58±10 years
Sex (M/F)	16/98
DAS28	5.7±1.2, n=114
CRP, mg/mL	2.7 (1.9–10.3), n=114
ESR, mm/h	36 (28–58), n=114
RF, U/mL	56 (20–198), n=107
Anti-CCP antibodies, U/mL	140 (54–246), n=67

**Figure 1:**
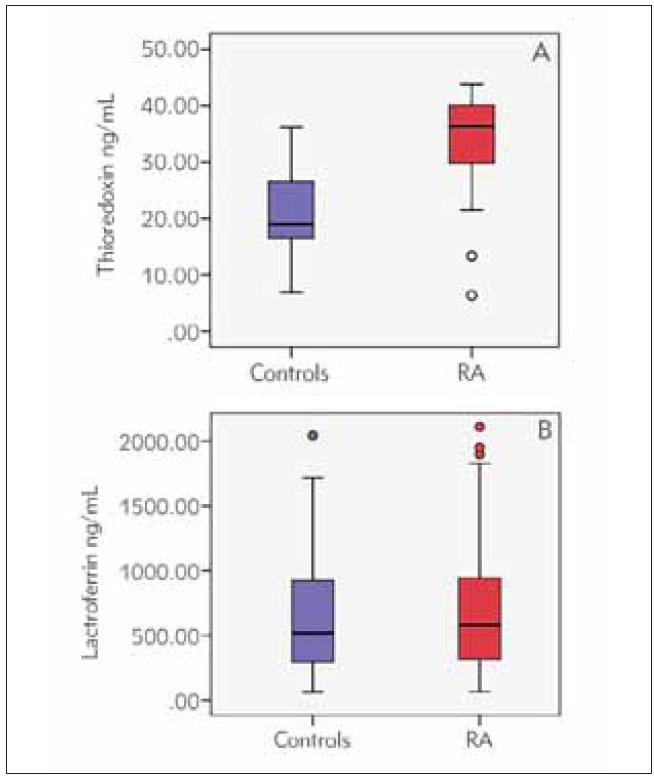
Serum levels of thioredoxin 1 and lactoferrin in healthy controls (n=21) and RA patients (n=114 for Trx, n=59 for Lf). The Mann-Whitney U test compares the control and patient groups, p<0.0001 (Figure 1A) and p=0.645 (Figure 1B).

Serum thioredoxin 1 concentration was determined in 59 RA patients at 36.4 (29.6–40.2) ng/mL. Serum lactoferrin concentration was determined in 114 RA patients at 579.6 (312.8–947.5) ng/mL. Serum thioredoxin 1 in 21 controls was 19.0 (16.3–26.8), ng/mL and serum lactoferrin in 39 controls was 519.0 (262.5–928.0), ng/mL. The statistical analysis of these results showed that serum thioredoxin 1 levels in RA were significantly higher compared to controls, p < 0.0001. Serum lactoferrin levels were elevated in RA compared to controls, but the difference was not statistically significant (Figure 1).

**Figure 2:**
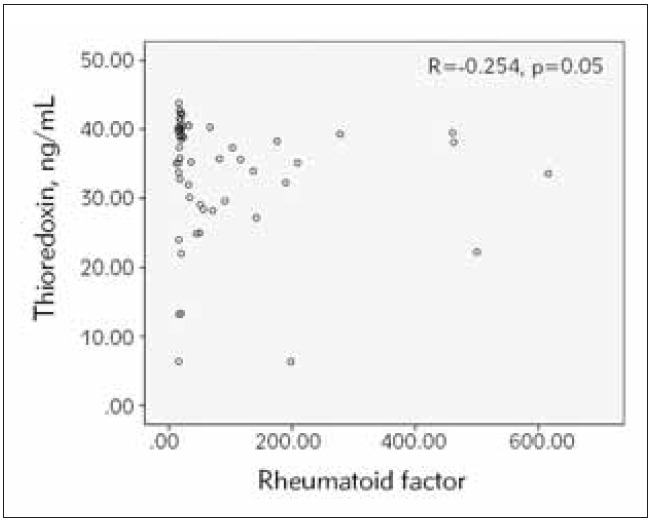
Correlation between serum thioredoxin 1 and rheumatoid factor in RA patients. (n=59, Spearman r =-0254, p=0.05).

**Figure 3:**
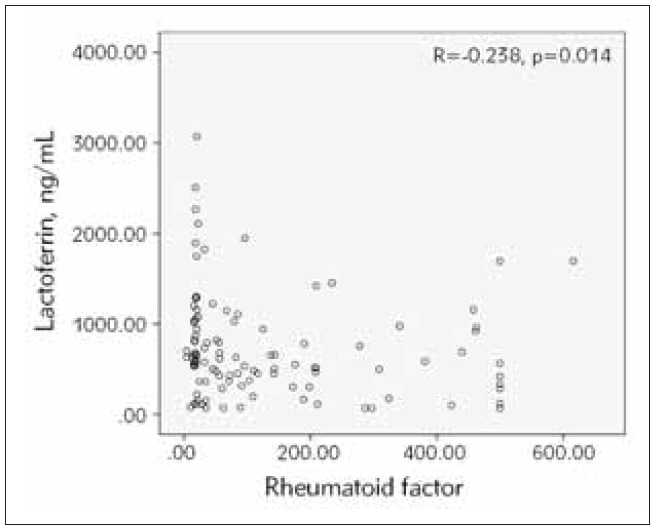
Correlation between serum lactoferrin and rheumatoid factor in RA patients. (n=114, Spearman r=-0.238, p=0.014).

The RA patients were distributed in groups according to sex, CRP levels and DAS28. There was no statistical difference in Trx 1 and Lf levels between males and females and between patients with normal and increased CRP and patients with DAS28<5.1 and DAS>5.1. Significant negative correlations of thioredoxin 1 and lactoferrin with rheumatoid factor were observed in RA patients, r=-0.254, p=0.05 and r=-0.238, p=0.014, respectively (Figure 2 and Figure 3). These negative correlations were observed also in females of RA patients, r=-0.278, p=0.048 and r=-0.276, p=0.007, respectively. The negative correlation of lactoferrin with rheumatoid factor was statistically significant also in the subgroup of patients with moderate disease activity (r=-0.352, p=0.038) and in the subgroup of patients with normal CRP (r=-0.329, p=0.005).

Trx 1 and Lf correlations were not statistically significant in RA patients with other disease markers, such as anti-CCP antibodies, DAS28, ESR, CRP and IL-6.

**Figure 4:**
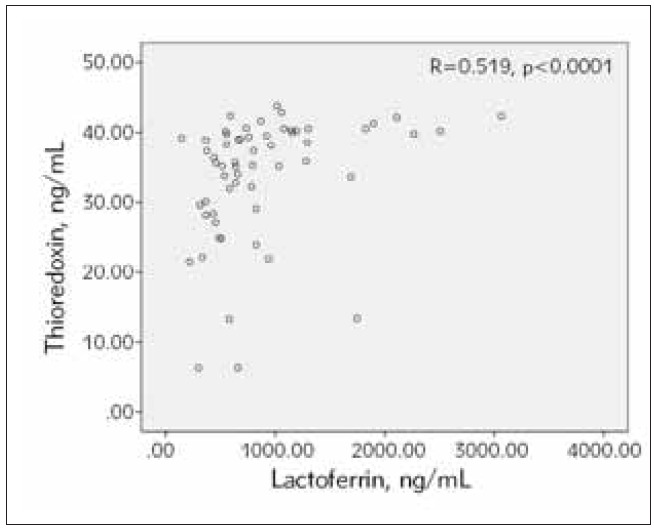
Correlation between serum thioredoxin 1 and lactoferrin in RA patients (n=59 Spearman r=0.519, p= <0.0001).

A strong, significant positive correlation between Trx and Lf was observed in the group of RA patients, r=0.519, p<0.0001 (Figure 4). This correlation was not significant in the control group, r=- 0.294, p=0.197. The association between Trx and Lf in RA patients was also significant in males (r=0.857, p=0.014) and females (r=0.508, p<0.0001), in the subgroups with DAS28<5.1 (r=0.722, p<0.0001) and DAS28>5.1 (r=0.350, p=0.046) and the subgroup with normal CRP level (r=0.522, p<0.0001).

## Discussion

In the present study, we investigated the serum levels of Trx 1 and Lf in patients with RA and healthy controls and established the association of these proteins with rheumatoid factor and other disease markers such as anti-CCP antibodies, DAS28, ESR, CRP and IL-6. We found that serum Trx 1 levels in RA patients were significantly higher when compared to controls (Figure 1). These findings agree with other authors who report increased levels of thioredoxin in the plasma or serum of rheumatoid arthritis patients [21] [22] [23] [24]. It is well known that the pathophysiology ofrheumatoid arthritis is associated with increased ROS production that initiates proinflammatory events and joint damage. The elevated levels of Trx 1 in RA can be explained by its increased expression required to counteract oxidative stress [22] [24]. A recent study has shown another link between thioredoxin and RA. The authors report that thioredoxin activates protein arginine deiminase (PAD) through non-covalent interactions, enhancing protein citrullination during RA [25].

We investigated whether serum Trx 1 reflected disease activity and inflammation in RA. The patients were divided into two groups according to DAS28 and CRP values. There was no statistically significant difference in Trx 1 levels between patients with moderate and high disease activity and between patients with normal and elevated CRP levels. Previous studies have reported that the redox Trx activity and Trx protein concentrations in serum correlated with DAS score, CRP and markers of oxidative stress, suggesting the potential use of Trx as a new biomarker reflecting the disease activity and the degree of oxidative stress in RA [21] [22].

To clarify the association between serum thioredoxin and disease markers in RA, we investigated the correlations of thioredoxin 1 with RF, anti-CCP antibodies, DAS28, ESR, CRP and IL-6. There was a significant negative correlation between Trx 1 levels and RF (Figure 2), suggesting a decrease in the active reduced Trx 1 with increased disease severity and progression and an association of Trx 1 with the pathological changes in RA. Circulating forms of Trx in the serum are mainly oxidized. When the protein is fully oxidized, it aggregates easily, and part of it cannot be recognized by ELISA antibodies [21].

Another multifunctional protein with antioxidant activity is the iron-binding protein lactoferrin, which can also suppress ROS generation and oxidative stress [17] [26]. In our study, serum Lf levels in RA patients were elevated compared to those in the control group. However, the difference was not statistically significant. Our results agree with those of other authors who report serum Lf levels in RA patients compared to healthy individuals [27] [28]. A previous study reported that synovial fluid Lf was significantly higher in RA patients than serum Lf. However, no correlation was found between synovial fluid Lf and serum Lf in RA patients [27]. It has been reported that neutrophil granulocytes are the predominant cell type in the synovial fluid of affected joints. Lf can serve as a marker of neutrophil activation due to its release at the site of inflammation [29] [30] [31].

The present study found that serum Lf in RA patients was inversely associated with rheumatoid factor (Figure 3). However, there was no significant correlation between Lf and other disease indicators such as anti-CCP antibodies, DAS28, ESR, CRP, and IL-6. A possible explanation of the negative association of Lf with RF is the depletion of Lf with the increase of RF due to the formation of anti-lactoferrin autoantibodies (ANCAs). ANCAs against myeloperoxidase (MPO), proteinase 3 (PR-3), lactoferrin (Lf), cathepsin G (CG) and elastase (EL) have been described. Among the five ANCAs, anti-Lf antibody (anti-Lf) was most commonly observed in patients with RA [32] [33]. It has been suggested that ANCAs occur especially frequently in RA patients who have longstanding, severe disease and who are positive for rheumatoid factor [33].

On the other hand, a recent study reports the ability of lactoferrin-containing IgG immunocomplex (Lf-IC) to drive the conversion of M2 into an M1-like phenotype of human macrophages. It was found that Lf-ICs were elevated in the serum of patients with rheumatoid arthritis and led to the activation of human monocytes/macrophages and inflammatory pathways via synergistic signalling through CD14/toll-like receptor (TLR) 4 and Fc RIIa (CD32a), triggering the production of proinflammatory cytokines IL-1, IL-6, and TNF-α [34]. Lf-IC-primed M2 cells promote the activation of memory Th17 cells, which are known to play a key role in RA pathology. The authors suggest that immunocomplexes between autoantibodies and biologically active autoantigens like lactoferrin can drive M2-M1 polarization and induce rheumatoid factor production by B-cells as a response [34] [35]. Therefore, the inverse association between lactoferrin and rheumatoid factor can also be explained by the engagement of lactoferrin in Lf-ICs, which influences the precise detection of free Lf in serum via immunoassay (ELISA).

Our findings support previous studies that describe Lf as a marker of neutrophil granulocyte activation rather than a disease activity marker in RA. Lactoferrin correlated with neutrophil granulocyte count but did not correlate with disease activity [29] [30]. The current study’s findings do not support previous studies in which plasma lactoferrin in RA patients correlated significantly with CRP [36] [37].

Further analysis of our results showed a significant positive correlation between Trx 1 and Lf in RA patients (Figure 4). The correlation was also observed in male and female patients, in the subgroups according to DAS28 value and in the subgroup of RA patients with normal CRP levels. The correlation was not significant in the control group. This finding suggests that thioredoxin 1 and lactoferrin levels increase simultaneously in response to the increased oxidative stress and disease progression. To control ROS formation, the body’s protective mechanisms use various enzymatic and non-enzymatic antioxidants [26]. A previous study reports that markers of oxidative stress correlate with oxidative tissue damage, disease activity (DAS28) and TNF-α and concludes that the measurement of oxidative stress in peripheral blood and/or synovial fluid could be an effective biomarker for monitoring disease activity in RA [31]. Thioredoxin 1 and lactoferrin are part of the body’s antioxidant defence and counteract oxidative tissue damage [26]. This is the first study reporting the inverse association of these proteins with the rheumatoid factor and the positive association between them in RA. Together, these results contribute to understanding the significance of Trx 1 and Lf levels to the pathogenesis of RA.

In conclusion, our study has established that thioredoxin 1 levels are significantly elevated in the serum of RA patients compared to controls. Thioredoxin 1 and lactoferrin correlate with rheumatoid factor but not with anti-CCP antibodies and systemic disease activity. Therefore, the two proteins may serve as new biomarkers for assessing pathological changes and monitoring disease severity and progression in RA.

## Dodatak

### Ethics approval and informed consent

The research was approved by the Ethics Committee at the Medical University of Plovdiv and conducted following the Declaration of Helsinki. Participation in the study was voluntary, and all subjects gave their informed consent for inclusion.

### Funding

This study was supported by an Intrauniversity research project number HO-11/2013 of the Medical University of Plovdiv.

### Conflict of interest statement

All the authors declare that they have no conflict of interest in this work.

### List of abbreviations

anti-CCP antibodies – anti-cyclic citrullinated peptide antibodies;<br>Lf – lactoferrin;<br>RA – rheumatoid arthritis;<br>RF – rheumatoid factor;<br>Trx – thioredoxin.
